# GPIHBP1 as a Biomarker of Diabetic Polyneuropathy and Vascular Complications in Type 2 Diabetes Mellitus

**DOI:** 10.3390/biom16050707

**Published:** 2026-05-11

**Authors:** Savelia Yordanova, Antoaneta Gateva, Diana Nikolova, Julieta Hristova, Zdravko Kamenov

**Affiliations:** 1 Department of Internal Medicine, Faculty of Medicine, Medical University of Sofia, 1431 Sofia, Bulgaria; agateva@medfac.mu-sofia.bg (A.G.); diananikolova@medfac.mu-sofia.bg (D.N.); zkamenov@medfac.mu-sofia.bg (Z.K.); 2 Department of Clinical Laboratory, Faculty of Medicine, Medical University of Sofia, 1431 Sofia, Bulgaria; jhristova@medfac.mu-sofia.bg

**Keywords:** GPIHBP1, diabetes mellitus, diabetic polyneuropathy

## Abstract

Background: Diabetic neuropathy is one of the most common chronic complications of diabetes mellitus and could lead to foot ulcerations, lower-limb amputations, increased mortality and reduced quality of life. This study examines the level of GPIHBP1 to assess its diagnostic and prognostic values across the metabolic continuum. Methods: This is an observational monocentric study, including 160 patients with type 2 diabetes mellitus, obesity without carbohydrate metabolism disorders and healthy controls. Clinical data and laboratory results were collected, and serum levels of GPIHBP1 were measured using an ELISA. The presence of DPN for the diabetes group was assessed using corneal confocal microscopy and NDS. The statistical analyses included *t*-tests, Pearson’s correlation analysis, and ROC analysis to explore associations and the predictive values of the biomarker. Results: The GPIHBP1 levels increased progressively, with the lowest levels observed in the control group, higher levels in patients with obesity, and the highest levels in those with diabetes mellitus. Higher GPIHBP1 levels were observed in patients with peripheral diabetic neuropathy compared to those without. GPIHBP1 demonstrated moderate discriminative performance for the presence of diabetes, diabetic neuropathy and nephropathy. GPIHBP1 levels were also associated with renal function parameters and markers of vascular involvement. After adjustment for confounders, including estimated glomerular filtration rate (eGFR), the association between GPIHBP1 and diabetic neuropathy remained statistically significant although attenuated. Higher levels were observed in patients with coronary artery disease, and a positive correlation was established with mean IMT and sudomotor dysfunction score. Conclusions: Circulating GPIHBP1 levels are associated with diabetes mellitus and its micro- and macrovascular complications, particularly diabetic neuropathy. Its measurement could enhance early diagnosis and personalized management of T2DM, and, while these findings support a potential role of GPIHBP1 as a biomarker of metabolic and vascular dysfunction, its clinical utility requires confirmation in longitudinal studies.

## 1. Introduction

Diabetic neuropathy (DN) is one of the most frequent chronic complications of diabetes mellitus, along with diabetic retinopathy, diabetic foot disease, and diabetic cardiovascular complications. As the number of diabetic patients continues to increase worldwide, diabetic peripheral polyneuropathy (DPN) has become a global health challenge [1]. DPN develops in about 50% of all people with diabetes, about half of whom develop neuropathic pain. Why half develop pain and the other half do not is not clear but almost certainly involves pathoanatomical and physiological differences as well as genetic and psychosocial reasons, similar to other chronic pain conditions [2]. Symptoms due to DPN typically comprise neuropathic pain, paresthesia, dysesthesia, and numbness in the distal lower limbs. On the other hand, asymptomatic DPN may affect up to 50% of patients with this condition. Distal symmetric diabetic polyneuropathy, foot ulcerations, and lower-limb amputation greatly increase mortality, which is 23% 2 years after a foot ulceration and rises to 71% after 10 years [3].

The current understanding of the mechanisms involved in DPN is largely based on aberrant glucose metabolism. Hyperglycemia-induced oxidative stress and reactive oxygen species result in peripheral nerve injury. Experimental data have demonstrated nitro-oxidative stress in dorsal root ganglia, axons and Schwann cells, with nerve conduction impairment, neurovascular dysfunction, apoptosis and sensory deficits [4].

Extensive research has demonstrated that oxidative stress is the common denominator linking the four major destructive pathways of hyperglycemia, including increased hexosamine pathway flux, activation of the protein kinase-C (PKC) pathway, increased advanced glycation end-product (AGE) formation, and increased polyol pathway flux [5]. These mechanisms are assumed to individually or synergistically trigger the onset and progression of DPN [6].

DPN is also associated with hyperlipidemia, insulin resistance and protein catabolism. The incidence of DPN is strongly associated with hyperglycemia, as well as with cardiovascular risk factors such as elevated cholesterol and triglyceride levels, hypertension, obesity, and smoking. These risk factors are amenable to modification through early intervention. Furthermore, factors such as obesity and hypertriglyceridemia have been identified as significant independent risk factors for DPN in individuals with T2D, irrespective of glycemic control [4].

Therapeutic approaches targeting a specific mechanism, such as those utilizing aldose reductase inhibitors or advanced glycation end-product inhibitors, have met with limited success, and normoglycemia is now generally accepted as the primary approach to the prevention of diabetic neuropathy. However, it is not achievable in a considerable number of patients. Clearly, designing effective treatments for diabetic neuropathy remains challenging, while its pathogenesis is still not completely understood. This highlights the need to identify additional biomarkers and pathogenic pathways that could improve both disease characterization and therapeutic targeting.

GPIHBP1 (glycosylphosphatidylinositol-anchored high-density lipoprotein-binding protein 1) is a protein found in the endothelial cells of capillaries that is anchored by glycosylphosphatidylinositol and binds to high-density lipoproteins. GPIHBP1 attaches to lipoprotein lipase (LPL), subsequently carrying the enzyme and transferring it to the capillary lumen. Enabling lipid metabolism is essential for the marginalization of lipoproteins alongside capillaries [7]. GPIHBP1 is expressed highly in the heart, adipose tissue, and skeletal muscle, the same tissues that express high levels of lipoprotein lipase (LPL). In each of these tissues, GPIHBP1 is located in capillary endothelial cells, serving as a protein partner to LPL [8].

The discovery of GPIHBP1 and its role in triglyceride metabolism has shown that GPIHBP1 transports LPL to capillaries, where it binds and stabilizes LPL, enabling the processing of triglyceride-rich lipoproteins (TRLs). Deficiency or mutations in GPIHBP1 mislocalize LPL, impair TRL processing, and can cause severe lifelong hypertriglyceridemia (chylomicronemia), leading to an increased risk of acute pancreatitis, underscoring the importance of GPIHBP1 in intravascular TG processing [9].

Mice that were deficient in glycosylphosphatidylinositol-anchored high-density lipoprotein-binding protein 1 (GPIHBP1) exhibited a striking accumulation of chylomicrons in the plasma and had plasma triglyceride levels in excess of 1000 mg/dL (11 mmol/L) without altered LPL expression in adipocytes and myocytes. Furthermore, the vast majority of the plasma triglycerides were located in the large lipoprotein fraction, suggesting a defect in lipolytic processing of chylomicrons. Further studies revealed that GPIHBP1 expression in capillary endothelial cells is critical for the transportation of LPL from the basolateral to the capillary apical surface [10].

In diabetes, the impairment of insulin secretion and insulin resistance contribute to hypertriglyceridemia as the enzymatic activity of lipoprotein lipase (LPL) depends on insulin action. While insulin increases LPL activity in the adipose tissue, physiological concentrations of insulin decrease the muscle LPL in proportion to the effect of insulin on muscle glucose uptake [11]. A study by Kurooka et al. proposed that GPIHBP1 levels are not altered in type 2 diabetes patients with higher serum triglyceride levels, whereas they are elevated in type 2 diabetes patients with diabetic retinopathy and nephropathy. The circulating regulators of the lipolytic complex might be new biomarkers for lipid and glucose metabolism and diabetic vascular complications [12]. The GPIHBP1 levels also changed rapidly, increasing during fasting when LPL activity decreased, although GPIHBP1 is known to stabilize LPL. The plasticity of the LPL system is severely blunted or completely lost in insulin-resistant rats [13]. In another study by Nagasawa et al., individuals with greater insulin resistance presented with lower LPL levels and increased GPIHBP1 levels, likely compensating for decreased LPL levels due to insulin resistance [14].

In conclusion, diabetes, insulin resistance and impaired insulin secretion reduce lipoprotein lipase (LPL) activity, contributing to hypertriglyceridemia. GPIHBP1, a key regulator of the lipolytic system, has been shown to remain unchanged in hypertriglyceridemia but to increase in patients with diabetic microvascular complications, such as retinopathy and nephropathy. Moreover, its dynamic regulation in response to metabolic states and its elevation in insulin resistance, despite reduced LPL levels, suggest a compensatory role in maintaining lipolytic function.

To date, limited data exist regarding the role of GPIHBP1 in diabetic polyneuropathy. While previous studies have focused mainly on nephropathy and retinopathy, its association with peripheral and autonomic neuropathy remains largely unexplored. Based on these observations, the aim of the present study was to investigate whether GPIHBP1 reflects underlying metabolic and microvascular disturbances and whether it could serve as a potential biomarker of diabetic polyneuropathy and other vascular complications in diabetes.

## 2. Materials and Methods

### 2.1. Study Design

A total of 160 adults (mean age 55.8 ± 8.7 years) were included in this single-center cross-sectional investigation. Participants were categorized into three groups: 93 patients with type 2 diabetes and 36 individuals with obesity and normal glucose metabolism and a healthy control group, consisting of 31 participants.

Eligibility required participants to be 18 years or older. Patients with type 2 diabetes mellitus were diagnosed according to American Diabetes Association criteria. The obesity group included individuals with elevated body mass index but without carbohydrate metabolism disorders, while the control group consisted of metabolically healthy individuals with normal BMI and glucose parameters. Individuals were not eligible for inclusion in the presence of any acute inflammatory or infectious disease, malignancy, advanced liver disease, end-stage renal failure, alcohol abuse, or other known causes of peripheral or autonomic neuropathy, as well as concomitant ocular diseases (keratoconus, glaucoma, etc.).

The study was conducted in accordance with the Declaration of Helsinki and was approved by the Ethics Committee of the Medical University of Sofia (Protocol No. 11/11 July 2023). Written informed consent was obtained from all participants prior to enrollment.

### 2.2. Clinical and Anthropometric Assessment

All participants underwent a standardized clinical assessment. Anthropometric measurements included body weight (kg), body mass index (BMI, kg/m^2^), waist circumference (cm), waist-to-hip ratio (WHR), and waist-to-stature ratio (WSR).

Cardiometabolic risk factors including arterial hypertension, dyslipidemia, smoking status, and metabolic syndrome were documented as well.

### 2.3. Diagnosis of Neuropathy

All assessments were performed in the morning after at least 12 h of fasting and abstinence from caffeine, alcohol, and medications affecting cardiovascular function following a resting period of 15–20 min.

### 2.4. Clinical Neuropathy Assessment (NDS)

Neurological evaluation of peripheral nerve function was assessed using a neuropathy disability score (NDS) that is modified for lower limbs. This assessment incorporated testing of pinprick perception (using a disposable neurotip), temperature discrimination (using warm/cold stimulus), vibration sensitivity (using a 128 Hz-tuning fork, applied to the hallux) and ankle reflex responses. Each sensory modality (vibration, temperature, and pinprick) was evaluated bilaterally and graded as present (normal) (0) or absent/reduced (1) for each foot (maximum 2 points per modality). Ankle reflexes were assessed on a three-point scale: 0 = normal, 1 = present with reinforcement, or 2 = absent for each side. The cumulative score ranged from 0 to 10, with values exceeding 5 considered indicative of greater and clinically significant neuropathic impairment [15].

### 2.5. Peripheral Neuropathy Assessment by Corneal Confocal Microscopy (CCM)

Small fiber involvement was further assessed using corneal confocal microscopy (Heidelberg Retinal Tomograph III with Rostock Cornea Module, Heidelberg Engineering, Heidelberg, Germany). For each participant, six high-resolution images of the central corneal subbasal nerve plexus (three per eye) were obtained and analyzed. Quantitative analysis included corneal nerve fiber density (CNFD)—fibers per mm^2^; corneal nerve branch density (CNBD)—branches per mm^2^; corneal nerve fiber length (CNFL)—total fiber length per mm^2^. Image processing was performed using automated software (ACCMetrics, version 2.0) in order to minimize observer bias. Interpretation of corneal nerve parameters was based on previously established normative reference values by Malik et al. [16].

### 2.6. Autonomic Neuropathy Assessment

Cardiovascular autonomic function was assessed under controlled resting conditions using a Cardiosys Extra system. Evaluation was performed according to Ewing’s cardiovascular reflex tests, following established consensus criteria. In our study, a total score of 3 or higher was considered consistent with the presence of autonomic neuropathy [17].

Participants fulfilling criteria for both peripheral and autonomic neuropathy were categorized as having combined neuropathy.

### 2.7. Sudomotor Function

Sudomotor function was evaluated using (Sudoscan^®^, Impeto Medical, Saint-Denis, France), which determines the electrochemical skin conductance (ESC) values from hands and feet. This method provides an indirect measure of sympathetic sudomotor activity and small fiber function. Risk categories were defined as low < 25%, moderate 25–49% and high ≥ 50% [18].

### 2.8. Assessment of Diabetic Nephropathy

Diabetic kidney disease was defined as the presence of albuminuria and/or reduced eGFR.

### 2.9. Laboratory Investigations

Venous blood samples were collected in the fasting state in EDTA-containing tubes. GPIHBP1 concentrations were measured using a commercially available ELISA kit (IBL International GmbH, Hamburg, Germany) and expressed in pg/mL. The ELISA assay was performed according to the manufacturer’s instructions. Plasma samples were stored at −80 °C until analysis and were thawed only once prior to measurement. All samples were analyzed in duplicate. The intra-assay and inter-assay coefficients of variation were <10% and <12%, respectively, as reported by the manufacturer.

### 2.10. Statistical Analysis

Analysis was conducted using the SPSS statistical software (version 23). Continuous variables are presented as mean ± standard deviation. Normality of data distribution was assessed using the Kolmogorov–Smirnov test. Variables with a normal distribution were analyzed using parametric methods, specifically analysis of variance (ANOVA). For variables that did not meet the assumptions of normality, nonparametric methods were applied. In particular, comparisons between two independent groups were performed using the Mann–Whitney U test. Correlation analyses were performed using Pearson correlation for normally distributed variables and Spearman’s rank correlation for non-normally distributed data. Multivariable logistic regression analysis was conducted to assess independent associations between variables. Variables included in the models were selected based on clinical relevance and univariate analysis, and potential multicollinearity was considered during model building. A *p*-value of less than 0.05 was considered statistically significant. No correction for multiple comparisons was applied due to the exploratory nature of the study.

## 3. Results

**Figure 1:**
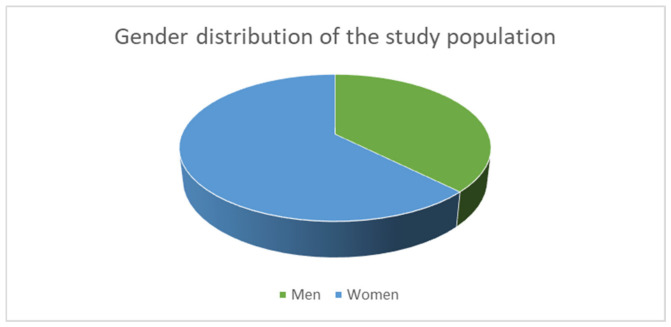
Gender distribution of the study population.

The study enrolled 160 participants with a mean age of 55.8 ± 8.7 years, divided into the following groups: Group 1 consisted of 93 patients with type 2 diabetes mellitus, Group 2 consisted of 36 individuals with obesity without carbohydrate metabolism disorders, and Group 3 comprised 31 controls with normal body weight. Further, 37.5% (*n* = 60) of the participants were male and 62.5% (*n* = 100) were female (Figure 1). Of the women studied, 79 (79%) were postmenopausal.

Patients with type 2 diabetes and those with obesity had similar weight and BMI, although the obese patients were younger. The controls were of similar age to the patients with diabetes but had lower weight and BMI compared to the other two groups (Table 1).

**Figure 2:**
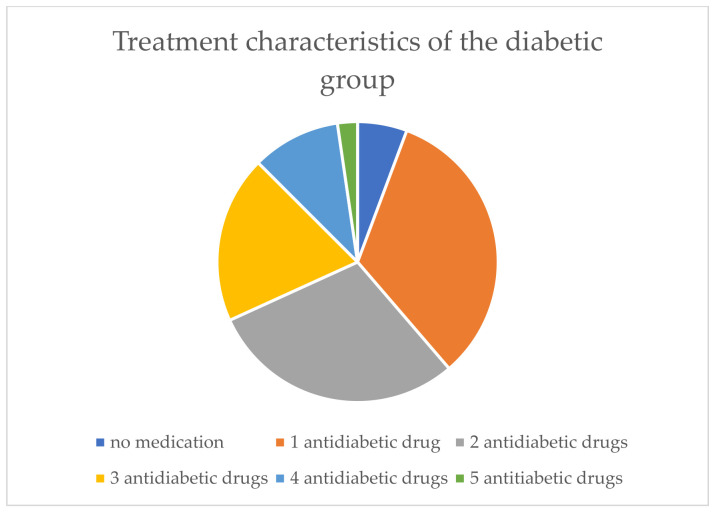
Treatment characteristics of the group (Group 1) presenting with diabetes mellitus type 2.

The average duration of diabetes in Group 1 was 8.86 years. Among the included patients with diabetes at the time of the study, 5.7% were not receiving antidiabetic therapy, 33% were on one antidiabetic medication, 29.5% on two, 19.3% on three, 10.2% on four, and 2.3% on five antidiabetic medications. The treatment characteristics are shown in Figure 2.

Further, 77.4% of the patients were on metformin, 36.3% on sulfonylureas, 12% on DPP-4 inhibitors, 20.7% on GLP-1 agonists, 25.8% on SGLT-2 inhibitors, and 21.7% on insulin among the studied patients with diabetes.

**Figure 3:**
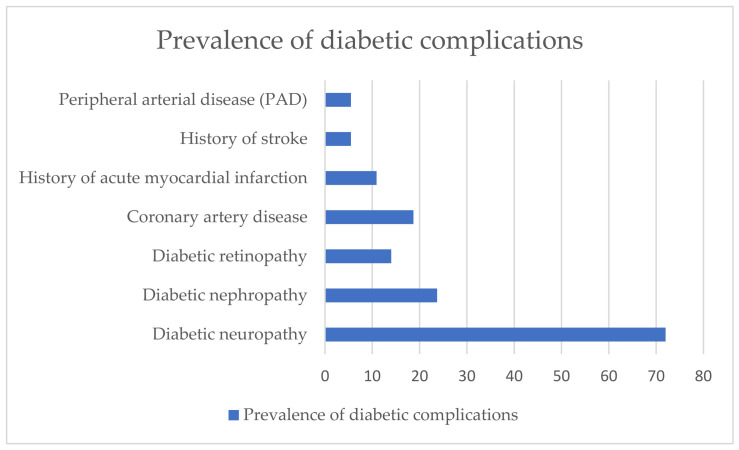
Diabetic complications in Group 1.

The diabetic complications are presented in Figure 3. Diabetic neuropathy was found in 72% of the patients with diabetes, diabetic nephropathy in 23.7%, diabetic retinopathy in 14%, coronary heart disease in 18.7%, with 10.9% having a history of acute myocardial infarction, 5.5% a history of stroke, and peripheral arterial disease in 5.5% of patients.

### 3.1. GPIHBP1 Levels Between Study Groups

The GPIHBP1 levels increased progressively, with the lowest levels observed in the control group, higher levels in patients with obesity, and the highest levels in those with diabetes mellitus (Table 2). When we further stratified the participants into those with diabetes and those without carbohydrate metabolism disorders (obese individuals and controls), we again found significantly higher levels in the diabetic group (Table 3).

### 3.2. GPIHBP1 Levels and Diabetic Complications

The GPIHBP1 levels were significantly higher in patients with peripheral diabetic neuropathy (established using corneal confocal microscopy) compared to those without (Table 4). No differences were found between patients with and without autonomic neuropathy.

The patients with diabetic nephropathy had significantly higher GPIHBP1 levels compared to those without (1315.5 ± 874.7 vs. 997.4 ± 328.0 pg/mL, *p* = 0.004). Similar results were observed in patients with coronary heart disease (1334.5 ± 1010.8 vs. 1013.8 ± 320.8 pg/mL, *p* = 0.01).

Postmenopausal patients had significantly higher GPIHBP1 levels (1021.1 ± 354.4 vs. 853.7 pg/mL, *p* = 0.025).

GPIHBP1 showed a weak positive correlation with the age of the study participants (r = 0.220, *p* = 0.005), GGT (r = 0.451, *p* < 0.001), serum creatinine (r = 0.333, *p* < 0.001), and a negative correlation with glomerular filtration rate (r = −0.363, *p* < 0.001). GPIHBP1 also correlated positively with the mean intima-media thickness in the carotid artery (r = 0.251, *p* = 0.03) and the sudomotor dysfunction score (ANR) (r = 0.211, *p* = 0.021).

**Figure 4:**
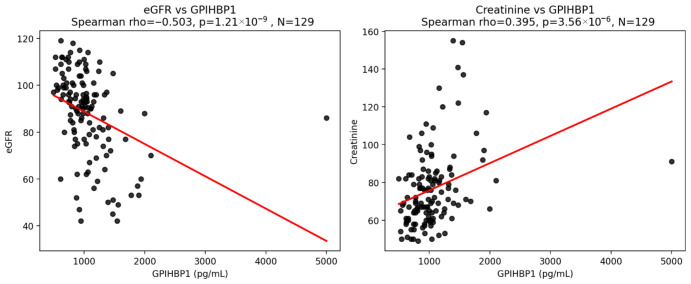
Correlation of circulating GPIHBP1 levels with estimated glomerular filtration rate (eGFR) and serum creatinine in the study population.

GPIHBP1 is very strongly associated with kidney function markers. It is negatively correlated with eGFR (rho = −0.503, *p* < 0.001) and positively correlated with creatinine (rho = 0.398, *p* < 0.001) (Figure 4).

No correlation was found with the duration of diabetes, visceral adiposity indices, systolic and diastolic blood pressure, lipid profile indices (including total cholesterol, Tg, LDL-C, VLDL and HDL-C), the oral glucose tolerance test, glycated hemoglobin, or ACR.

### 3.3. Predictive Value of GPIHBP1 in Diabetic Complications

**Figure 5:**
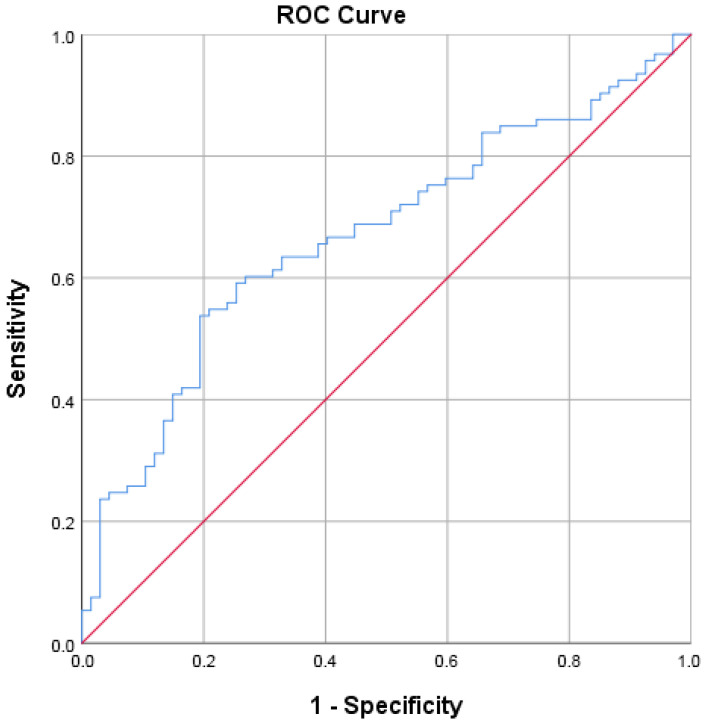
Receiver operating characteristic (ROC) curve of circulating GPIHBP1 for discrimination between patients with type 2 diabetes mellitus and non-diabetic individuals (controls and obesity group). The area under the curve (AUC) reflects moderate discriminative ability.

GPIHBP1 showed moderate discriminative ability for the presence of diabetes mellitus (Figure 5 and Table 5).

**Figure 6:**
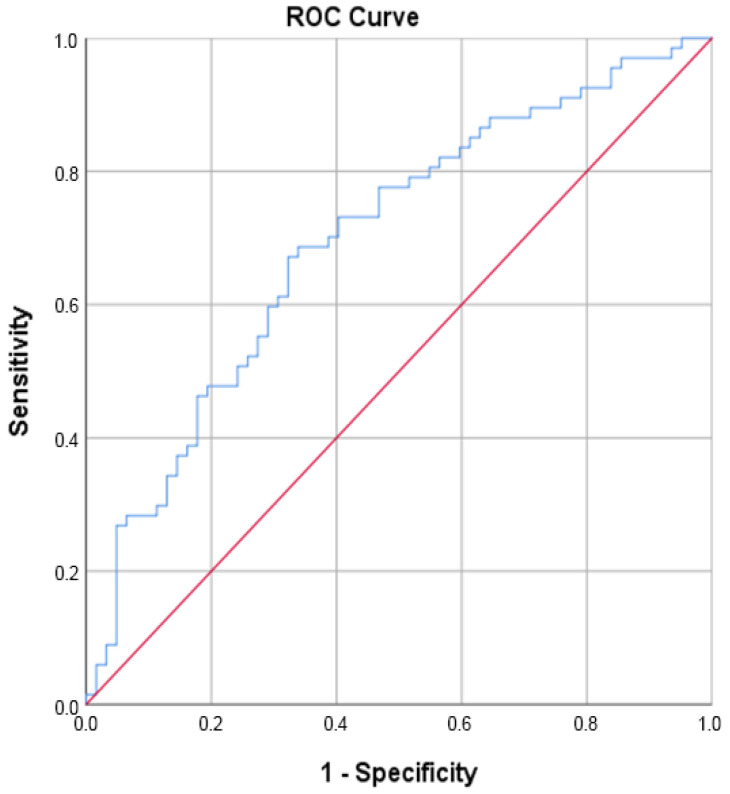
Receiver operating characteristic (ROC) curve of circulating GPIHBP1 for discrimination between patients with and without diabetic polyneuropathy. The AUC indicates moderate discriminative ability.

GPIHBP1 showed moderate discriminative ability for the presence of diabetic neuropathy (Figure 6 and Table 6).

**Figure 7:**
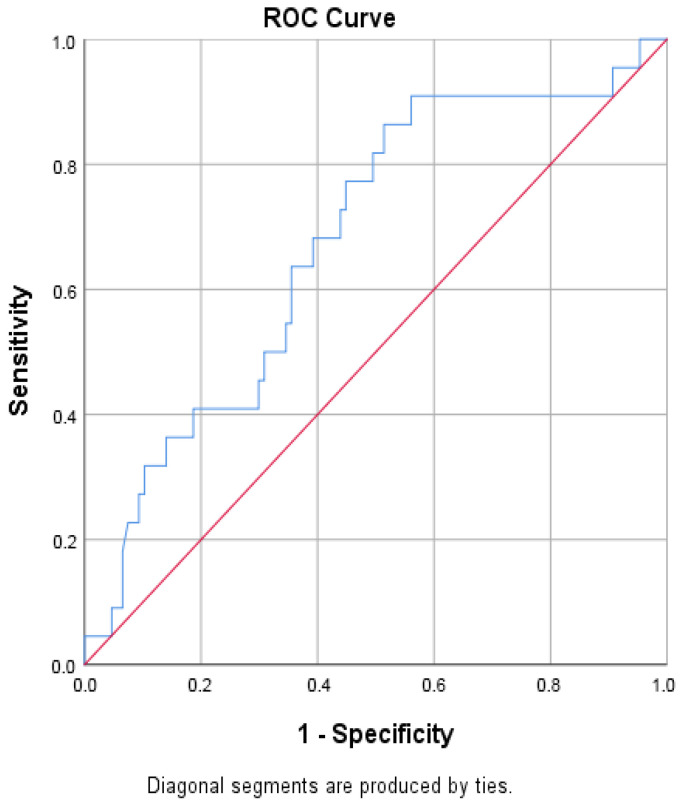
Receiver operating characteristic (ROC) curve of circulating GPIHBP1 for discrimination between patients with and without diabetic nephropathy. The AUC indicates moderate discriminative ability.

GPIHBP1 showed moderate discriminative ability for the presence of diabetic nephropathy (Figure 7 and Table 7).

**Figure 8:**
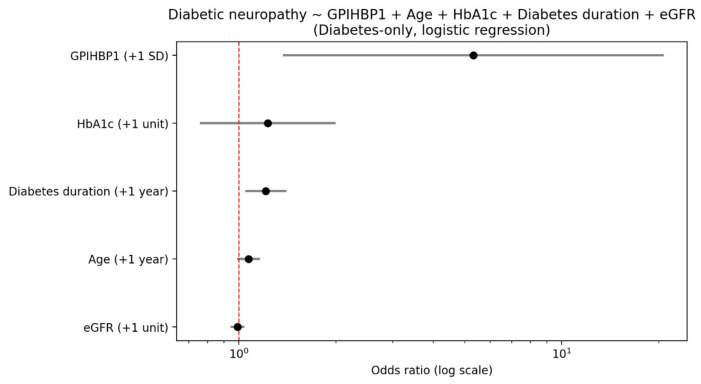
Logistic regression analysis of diabetic neuropathy in patients with diabetes. Odds ratios (ORs) and 95% confidence intervals (CIs) are shown for GPIHBP1, HbA1c, diabetes duration, age, and eGFR.

In the logistic regression model, after accounting for age/sex, higher GPIHBP1 is associated with higher odds of diabetic neuropathy (OR 2.02, 95% CI 1.03–3.97, *p* = 0.0406), while other complications and coronary heart disease do not show clear associations. When we selected only patients with diabetes and adjusted for age, sex, HBA1c and diabetes duration, GPIHBP1 was strongly associated with neuropathy even after controlling for duration and glycemic control (HbA1c); the odds ratio per +1 SD higher GPIHBP1 is 6.47 (CI 1.93–21.65; *p* = 0.0024). Within people who already have diabetes, higher GPIHBP1 is strongly associated with neuropathy even after controlling for duration and glycemic control (HbA1c) (Figure 8).

As GPIHBP1 is strongly correlated with renal function we also adjusted for eGFR. After adjusting for kidney function (eGFR), the association between GPIHBP1 and diabetic neuropathy was attenuated but remained statistically significant (OR = 5.32, CI (1.36–20.7), *p* = 0.015). A model comparison suggested that adding eGFR did not materially improve the model fit (LRT *p* = 0.68; AIC/BIC not improved), and discriminative ability was unchanged (AUC ~0.86).

## 4. Discussion

The present study demonstrates that circulating GPIHBP1 levels increase progressively throughout the metabolic spectrum, with the lowest levels observed in healthy controls, intermediate levels in individuals with obesity, and the highest levels in patients with type 2 diabetes mellitus. Importantly, this is, to our knowledge, one of the first studies to demonstrate an independent association between circulating GPIHBP1 levels and diabetic neuropathy, assessed using both corneal confocal microscopy and clinical scoring systems (NDS). This expands the current understanding of GPIHBP1 beyond its previously described associations with retinopathy and nephropathy.

Our results show that GPIHBP1 is strongly associated with diabetic microvascular complications, particularly diabetic neuropathy and nephropathy, and is independently associated with the presence of diabetes, diabetic nephropathy and diabetic neuropathy even after adjustment for age, sex, glycemic control and diabetes duration. Although many studies have focused on GPIHBP1 and diabetic nephropathy [19,20], associations with diabetic neuropathy remain unexplored, highlighting an important research gap for future investigation. It is important to mention that the present study was not specifically designed to evaluate sex-related differences. Although sex was included as a control variable, the sample size and sex distribution limited the ability to perform reliable stratified analyses. Future studies should investigate potential gender-specific effects.

The moderate but meaningful discriminative ability of GPIHBP1 for the presence of diabetes and its complications, particularly diabetic neuropathy, is intriguing [21]. A study by Kurooka et al. supports the present findings, demonstrating that higher levels of GPIHBP1 are observed in patients with diabetic retinopathy and nephropathy, strengthening its association with microvascular disruption. Similar to this study, we also found no correlation between rising levels of GPIHBP1 and the lipid profile of the patients. This further supports its role as a marker of microvascular damage and endothelial dysfunction rather than a direct cause of dyslipidemia. This is in line with previous evidence indicating that GPIHBP1 plays a key role in the transport and stabilization of lipoprotein lipase at the capillary level rather than serving as a marker of circulating lipid levels [10]. Another study by Miyashita et al. also found only a small inverse correlation between GPIHBP1 and triglyceride levels, which suggests that its variations do not reliably correlate with lipid levels [22].

The observed strong association between GPIHBP1 and renal function raises important questions regarding biomarker specificity. A study by Shenyang et al. [23] demonstrates that GPIHBP1 plays a critical role in renal lipid homeostasis and protection against tubular injury. These results suggest that renal dysfunction may influence circulating GPIHBP1 levels, either through reduced clearance or shared microvascular mechanisms. The attenuation of the association between GPIHBP1 and neuropathy after adjustment for eGFR indicates that kidney function may act as a partial confounder or mediator in this relationship. However, after the adjustment, the association between GPIHBP1 and diabetic neuropathy remained statistically significant although slightly attenuated. Importantly, model fit and discriminative performance were not materially improved by the inclusion of eGFR. These findings suggest that the relationship between GPIHBP1 and diabetic neuropathy is not solely driven by renal impairment but may reflect additional mechanisms, such as endothelial dysfunction and altered lipid transport. Vaziri et al. found that LPL deficiency in chronic kidney disease is associated with and compounded by GPIHBP1 deficiency, contributing to impaired clearance of triglyceride-rich lipoproteins, diminished availability of lipid fuel for energy storage in adipocytes and energy production in myocytes, and consequent hypertriglyceridemia, cachexia, muscle weakness and atherosclerosis [20]. This observation highlights the disruption of the GPIHBP1–LPL axis, which contributes to lipid abnormalities and renal dysfunction. More recent research has focused on the role of LPL as a key gene in diabetic kidney disease, with its expression tightly connected with immune-regulation effects in the progression of diabetic kidney disease [24].

The potential link to nerve damage is possibly related to mechanisms involving endothelial dysfunction and impaired lipid metabolism. Research by Bashir et al., focused on familial chylomicronemia syndrome, further highlights the GPIHBP1–LPL pathway in neural integrity as reduced or dysfunctional GPIHBP1 could lead to both sensory and autonomic neuropathy [25]. In contrast, our findings demonstrate elevated levels of GPIHBP1 in patients with DPN. The difference could reflect that, while in familial/genetic disorders there could be a loss of function in the protein, the different pathogenesis of T2D may indicate a compensatory response to metabolic stress, leading to impaired lipid utilization.

Our observed association between GPIHBP1 and ANR score further supports a link with sudomotor dysfunction, suggesting involvement of small nerve fibers and the autonomic nervous system.

Given the crucial role of GPIHBP1 in facilitating LPL transport and maintaining lipolytic activity, alterations in its levels could cause insulin resistance and diabetes mellitus. A study by Nyren et al. [26] found that both LPL and GPIHBP1 are present in mouse pancreas, and, although GPIHBP1 is not directly involved in pancreatic b-cell function, its levels could correlate with and reflect the metabolic and endothelial stress associated with IR and T2D. Our findings suggest very strong discriminative ability for the presence of diabetes mellitus, suggesting that GPIHBP1 may serve to identify high-risk individuals.

In the context of insulin resistance and T2D, reduced LPL activity is a well-established mechanism that contributes to lipid dysregulation [27,28]. The observed increased levels of GPIHBP1, however, could demonstrate a compensatory response aimed at preserving lipolytic function. In T2D, insulin resistance disrupts the activity of LPL, mainly in adipose tissue and the heart, leading to triglyceride accumulation and lipotoxicity. This disruption in lipid homeostasis forms the core of the development of atherosclerosis and diabetic cardiomyopathy [29]. Our results show significantly higher GPIHBP1 levels in patients with coronary artery disease compared to those without, as well as a positive correlation between GPIHBP1 and intima-media thickness. This supports the observation that elevated levels of GPIHBP1 could reflect early atherosclerotic changes and cardiovascular risk.

Lastly, circulating estrogen is a regulator of lipoprotein lipase (LPL). LPL catalyzes the hydrolysis of VLDL to form IDL and later LDL. After menopause, due to estrogen deficiency, there is increased plasma LPL and hepatic TG lipase activity, causing plasma LDL accumulation and also leading to down-regulation of LDL receptors [29]. Many studies have explored the relationship between menopause and metabolic dysregulation [30,31]. Our observation that postmenopausal patients had significantly higher GPIHBP1 levels suggests a compensatory mechanism to facilitate LPL transport to the capillary endothelium. These lipid alterations, together with estrogen deficiency, are closely linked to increased insulin resistance and a higher risk of developing type 2 diabetes, highlighting menopause as a critical period for altered carbohydrate metabolism.

Although the study provides insights into the relationship between GPIHBP1 and diabetes and its micro- and macrovascular complications, several limitations should be acknowledged. The cross-sectional design precludes conclusions regarding causality, and the sample size, although adequate, is relatively modest. Although statistically significant, the AUC values (~0.67–0.70) indicate moderate discrimination, suggesting limited standalone diagnostic utility. Finally, GPIHBP1 plays a key role in the transport and stabilization of lipoprotein lipase, but the present study did not include a direct assessment of LPL activity. Therefore, mechanistic interpretations regarding the GPIHBP1–LPL axis remain theoretical and should be interpreted with caution.

Future studies should focus on longitudinal assessment to determine whether GPIHBP1 predicts the onset and progression of diabetic neuropathy and other vascular complications. Larger multicenter cohorts are also needed to validate its diagnostic and prognostic performance and clinical utility. Experimental studies are also needed to elucidate the mechanistic link between GPIHBP1, lipid metabolism, and nerve damage. Ultimately, evaluating GPIHBP1 in combination with other biomarkers may improve early detection and risk stratification in patients with type 2 diabetes.

## 5. Conclusions

In conclusion, GPIHBP1 levels are elevated across the metabolic continuum, with the highest levels observed in type 2 diabetes. The association with diabetic nephropathy, particularly diabetic neuropathy, further supports its link to microvascular complications in diabetes. Moreover, the observed relationships between GPIHBP1, coronary artery disease, and IMT suggest involvement not only at the microvascular level but at the macrovascular level as well. Therefore, GPIHBP1 represents a candidate biomarker that reflects both metabolic and vascular dysfunction, including diabetic polyneuropathy and overall vascular risk in diabetes. Future longitudinal studies are needed to determine its potential role in risk stratification, disease progression, and clinical application.

## Figures and Tables

**Table 1:** Baseline characteristics of the groups.

Baseline Characteristics			
	Group 1 Diabetes Mellitus	Group 2 Obesity	Group 3 Controls
Age (y)	58.6 ± 8.2	49.2 ± 8.5 *	54.7 ± 5.9
Weight (kg)	98.2 ± 17.9	94.6 ± 16.3	66.0 ± 6.7 *
BMI (kg/m^2^)	34.9 ± 5.8	35.2 ± 3.8	23.9 ± 1.1 *

Group 1 (T2DM, *n* = 93); Group 2 (obesity, *n* = 36); Group 3 (controls, *n* = 31); * statistically significant difference (*p* < 0.05).

**Table 2:** Circulating GPIHBP1 levels in patients with type 2 diabetes mellitus, obesity, and healthy controls.

	Group 1 Diabetes Mellitus Type 2	Group 2 Obesity	Group 3 Controls
GPIHBP1 pg/mL	1095.3 ± 534.7	938.7 ± 260.9	796.6 ± 226.9 ^#^

Data are presented as mean +/− SD. ^#^ *p* < 0.05 between Group 1 and Group 3; Group 1 (T2DM, *n* = 93); Group 2 (obesity, *n* = 36); Group 3 (controls, *n* = 31).

**Table 3:** Circulating GPIHBP1 levels in participants with type 2 diabetes mellitus and individuals without carbohydrate metabolism disorders.

	Diabetes Mellitus Type 2	No Carbohydrate Abnormalities
GPIHBP1 pg/mL	1095.3 ± 534.7	872.9 ± 254.2 * (*p* = 0.002)

* *p* < 0.05. T2DM, *n* = 93; no carbohydrate abnormalities, *n* = 67.

**Table 4:** Circulating GPIHBP1 levels in patients with and without diabetic polyneuropathy.

	With Diabetic Neuropathy	Without Diabetic Neuropathy
GPIHBP1 pg/mL	1171.2 ± 574.9 (*p* = 0.003)	922.6 ± 300.5

With diabetic neuropathy, *n* = 67; without diabetic neuropathy, *n* = 26.

**Table 5:** ROC analysis of GPIHBP1 for discrimination of type 2 diabetes mellitus. The discriminative ability of GPIHBP1 was interpreted based on AUC values, with values of 0.6–0.7 considered moderate discrimination.

Area Under the Curve				
Test Result Variable(s): GPIHBP1 pg/mL				
Area	Std. Error ^a^	Asymptotic Sig. ^b^	Asymptotic 95% Confidence Interval	
			Lower Bound	Upper Bound
0.669	0.043	0.000	0.586	0.753

^a^. Under the nonparametric assumption. ^b^. Null hypothesis: true area = 0.5.

**Table 6:** ROC analysis of GPIHBP1 for discrimination of diabetic polyneuropathy. The discriminative ability of GPIHBP1 was interpreted based on AUC values, with values of 0.6–0.7 considered moderate discrimination.

Area Under the Curve				
Test Result Variable(s): GPIHBP1 pg/mL				
Area	Std. Error ^a^	Asymptotic Sig. ^b^	Asymptotic 95% Confidence Interval	
			Lower Bound	Upper Bound
0.697	0.046	0.000	0.607	0.788

^a^. Under the nonparametric assumption. ^b^. Null hypothesis: true area = 0.5.

**Table 7:** ROC analysis of GPIHBP1 for discrimination of diabetic nephropathy. The discriminative ability of GPIHBP1 was interpreted based on AUC values, with values of 0.6–0.7 considered moderate discrimination.

Area Under the Curve				
Test Result Variable(s): GPIHBP1 pg/mL				
Area	Std. Error ^a^	Asymptotic Sig. ^b^	Asymptotic 95% Confidence Interval	
			Lower Bound	Upper Bound
0.675	0.061	0.010	0.555	0.795

^a^. Under the nonparametric assumption. ^b^. Null hypothesis: true area = 0.5.

## Data Availability

The data presented in this study are available on request from the corresponding author due to ethical and privacy considerations.

## References

[1] Zhu J., Hu Z., Luo Y., Liu Y., Luo W., Du X., Luo Z., Hu J., Peng S. (2024). Diabetic peripheral neuropathy: Pathogenetic mechanisms and treatment. Front. Endocrinol..

[2] Zuidema X., de Galan B., Brouwer B., Cohen S.P., Eldabe S., Argoff C.E., Huygen F., Van Zundert J. (2024). 4. Painful diabetic polyneuropathy. Pain Pract..

[3] Bell D.S.H., Jerkins T.W. (2026). Diabetic distal symmetric polyneuropathy: More than just “tingling in the feet”. Diabetes Obes. Metab..

[4] Burgess J., Frank B., Marshall A., Khalil R.S., Ponirakis G., Petropoulos I.N., Cuthbertson D.J., Malik R.A., Alam U. (2021). Early Detection of Diabetic Peripheral Neuropathy: A Focus on Small Nerve Fibres. Diagnostics.

[5] Amara F., Hafez S., Orabi A., El Etriby A., Abdel Rahim A.A., Zakaria E., Koura F., Talaat F.M., Gawish H., Attia I. (2019). Review of Diabetic Polyneuropathy: Pathogenesis, Diagnosis and Management According to the Consensus of Egyptian Experts. Curr. Diabetes Rev..

[6] Mizukami H., Osonoi S. (2020). Pathogenesis and Molecular Treatment Strategies of Diabetic Neuropathy Collateral Glucose-Utilizing Pathways in Diabetic Polyneuropathy. Int. J. Mol. Sci..

[7] Jiang S., Ren Z., Yang Y., Liu Q., Zhou S., Xiao Y. (2023). The GPIHBP1-LPL complex and its role in plasma triglyceride metabolism: Insights into chylomicronemia. Biomed. Pharmacother..

[8] Beigneux A.P., Davies B.S., Bensadoun A., Fong L.G., Young S.G. (2009). GPIHBP1, a GPI-anchored protein required for the lipolytic processing of triglyceride-rich lipoproteins. J. Lipid Res..

[9] Fong L.G., Young S.G., Beigneux A.P., Bensadoun A., Oberer M., Jiang H., Ploug M. (2016). GPIHBP1 and Plasma Triglyceride Metabolism. Trends Endocrinol. Metab..

[10] Beigneux A.P., Davies B.S., Gin P., Weinstein M.M., Farber E., Qiao X., Peale F., Bunting S., Walzem R.L., Wong J.S. (2007). Glycosylphosphatidylinositol-anchored high-density lipoprotein-binding protein 1 plays a critical role in the lipolytic processing of chylomicrons. Cell Metab..

[11] Kiens B., Lithell H., Mikines K.J., Richter E.A. (1989). Effects of insulin and exercise on muscle lipoprotein lipase activity in man and its relation to insulin action. J. Clin. Investig..

[12] Kurooka N., Eguchi J., Wada J. (2023). Role of glycosylphosphatidylinositol-anchored high-density lipoprotein binding protein 1 in hypertriglyceridemia and diabetes. J. Diabetes Investig..

[13] Kroupa O., Vorrsjö E., Stienstra R., Mattijssen F., Nilsson S.K., Sukonina V., Kersten S., Olivecrona G., Olivecrona T. (2012). Linking nutritional regulation of Angptl4, Gpihbp1, and Lmf1 to lipoprotein lipase activity in rodent adipose tissue. BMC Physiol..

[14] Nagasawa T., Sakamaki K., Yoshida A., Machida H., Murakami F., Hashimoto M., Shinohara T., Murakami M., Tsunekawa K., Kimura T. (2025). Reciprocal Fluctuations in Lipoprotein Lipase, Glycosylphosphatidylinositol-Anchored High-Density Lipoprotein-Binding Protein 1, and Hepatic Triglyceride Lipase Levels in the Peripheral Bloodstream Are Correlated with Insulin Resistance. Nutrients.

[15] Young M.J., Boulton A.J., MacLeod A.F., Williams D.R., Sonksen P.H. (1993). A multicentre study of the prevalence of diabetic peripheral neuropathy in the United Kingdom hospital clinic population. Diabetologia.

[16] Tavakoli M., Ferdousi M., Petropoulos I.N., Morris J., Pritchard N., Zhivov A., Ziegler D., Pacaud D., Romanchuk K., Perkins B.A. (2015). Normative values for corneal nerve morphology assessed using corneal confocal microscopy: A multinational normative data set. Diabetes Care.

[17] Spallone V., Ziegler D., Freeman R., Bernardi L., Frontoni S., Pop-Busui R., Stevens M., Kempler P., Hilsted J., Tesfaye S. (2011). Cardiovascular autonomic neuropathy in diabetes: Clinical impact, assessment, diagnosis, and management. Diabetes Metab. Res. Rev..

[18] Cobuz C., Ungureanu-Iuga M., Anton-Paduraru D.T., Cobuz M. (2025). Possible Use of the SUDOSCAN Nephropathy Risk Score in Chronic Kidney Disease Diagnosis: Application in Patients with Type 2 Diabetes. Biosensors.

[19] Abass H.A., Baban R.S., shaker Khudair M. (2022). Functional impact of GPIHBP1 gene polymorphisms on the development of diabetic neuropathy in patients with type 2 diabetes mellitus. Chin. J. Med..

[20] Vaziri N.D., Yuan J., Ni Z., Nicholas S.B., Norris K.C. (2012). Lipoprotein lipase deficiency in chronic kidney disease is accompanied by down-regulation of endothelial GPIHBP1 expression. Clin. Exp. Nephrol..

[21] Pei-Ling Chiu A., Wang F., Lal N., Wang Y., Zhang D., Hussein B., Wan A., Vlodavsky I., Rodrigues B. (2014). Endothelial cells respond to hyperglycemia by increasing the LPL transporter GPIHBP1. Am. J. Physiol.-Endocrinol. Metab..

[22] Miyashita K., Fukamachi I., Nagao M., Ishida T., Kobayashi J., Machida T., Nakajima K., Murakami M., Ploug M., Beigneux A.P. (2018). An enzyme-linked immunosorbent assay for measuring GPIHBP1 levels in human plasma or serum. J. Clin. Lipidol..

[23] Li S., Nagothu K., Ranganathan G., Ali S.M., Shank B., Gokden N., Ayyadevara S., Megyesi J., Olivecrona G., Chugh S.S. (2012). Reduced kidney lipoprotein lipase and renal tubule triglyceride accumulation in cisplatin-mediated acute kidney injury. Am. J. Physiol.-Ren. Physiol..

[24] Dong Q., Xu H., Xu P., Liu J., Shen Z. (2025). Bioinformatic analysis identifies LPL as a critical gene in diabetic kidney disease via lipoprotein metabolism. Front. Endocrinol..

[25] Bashir B., Pasha R., Kamath A., Wang J., Malik R.A., Hegele R.A., Ferdousi M., Soran H. (2025). Neurodegeneration in familial chylomicronemia syndrome. J. Clin. Lipidol..

[26] Nyrén R., Chang C.L., Lindström P., Barmina A., Vorrsjö E., Ali Y., Juntti-Berggren L., Bensadoun A., Young S.G., Olivecrona T. (2012). Localization of lipoprotein lipase and GPIHBP1 in mouse pancreas: Effects of diet and leptin deficiency. BMC Physiol..

[27] Pruneta-Deloche V., Sassolas A., Dallinga-Thie G.M., Berthezène F., Ponsin G., Moulin P. (2004). Alteration in lipoprotein lipase activity bound to triglyceride-rich lipoproteins in the postprandial state in type 2 diabetes. J. Lipid Res..

[28] Gu J., Han X., Chen X., Lyu A., Cheung K.C.P. (2025). Cardiac Metabolomic Alterations in Diabetes: Interplay with Lipoprotein Lipase—A Systematic Review. Int. J. Mol. Sci..

[29] Swapnali R.K., Kisan R., Murthy D. (2011). Effect of menopause on lipid profile and apolipoproteins. Al Ameen J. Med. Sci..

[30] Lambrinoudaki I., Paschou S.A., Armeni E., Goulis D.G. (2022). The interplay between diabetes mellitus and menopause: Clinical implications. Nat. Rev. Endocrinol..

[31] Choi M.J., Yu J. (2025). Menopause and Diabetes Risk Along with Trajectory of β-Cell Function and Insulin Sensitivity: A Community-Based Cohort Study. Healthcare.

